# ORALLY ACTIVE, CLINICALLY TRANSLATABLE SENOLYTICS RESTORE Α-KLOTHO IN MICE AND HUMANS

**DOI:** 10.1093/geroni/igac059.2660

**Published:** 2022-12-20

**Authors:** Yi Zhu, Larissa Langhi Prata, Erin Wissler Gerdes, Jair Netto, Tamar Pirtskhalava, Nino Giorgadze, Utkarsh Tripathi, Christina Inman

**Affiliations:** Mayo Clinic, Rochester, Minnesota, United States; Mayo Clinic, Rochester, Minnesota, United States; Mayo Clinic, Rochester, Minnesota, United States; Mayo Clinic, Rochester, Minnesota, United States; Mayo Clinic, Rochester, Minnesota, United States; May Clinic, Rochester, Minnesota, United States; Mayo Clinic, Rochester, Minnesota, United States; Mayo Clinic, Rochester, Minnesota, United States

## Abstract

Decreased α-Klotho, a geroprotective factor, and increased senescent cell burden are both associated with early onset of physical disability, cognitive impairment, and premature all-cause mortality. It has been demonstrated that eliminating senescent cells can enhance physical function, cognition, and survival in mice, as does overexpressing α-Klotho. Mice with low α-Klotho exhibit accelerated senescent cell accumulation, recombinant α-Klotho decreases senescent cell burden and restores lifespan in these mice, and senescent epidermal cells are reduced in mice overexpressing α-Klotho. Here, we tested the hypothesis that senescent cells cause decreased α-Klotho and hence that reducing senescent cells can increase α-Klotho. Senescent cell conditioned medium (CM) reduced α-Klotho in cultured non-senescent human umbilical vein endothelial cells (HUVECs), renal tubular endothelial cells, and astrocytes. These effects of senescent CM were partially attenuated by neutralizing antibodies against the senescence-associated secretory phenotype (SASP) factors, activin A and IL-1α. Transplanting senescent cells into younger mice caused decreased urine and brain α-Klotho. Genetically reducing highly p16Ink4a-expressing cells in old INK-ATTAC mice or administering the senolytics, Dasatinib plus Quercetin (D+Q) or Fisetin (F), to young mice transplanted with senescent cells, young diet-induced obese (DIO) mice, or naturally-aged mice increased urine, kidney, and/or brain α-Klotho. Treating patients with idiopathic pulmonary fibrosis (IPF), a cellular senescence-related disease, with D+Q led to increased urinary α-Klotho. Thus, targeting senescent cells causes increases in the geroprotective factor α-Klotho, potentially amplifying the beneficial effects of senolytic drugs.

